# Supplementary material for: Awareness and compliance with tobacco control policies among retailers near schools in Arghakhanchi, Nepal: A mixed- methods study
Source: PLOS Glob Public Health. 2025 Jul 7;5(7):e0004780. doi: 10.1371/journal.pgph.0004780 (PMC12233269; doi:10.1371/journal.pgph.0004780)
Supplement: Attachment — Submitted filename: PAPER REVIEW- PLOS.docx [file pgph.0004780.s003.docx]

PAPER REVIEW- COMMENTS

Title: Awareness and compliance with tobacco control policies among retailers of nearby schools in Arghakhanchi district of Nepal: A mixed methods study

Short title: Consider above

Order of authors: Arjun Poudel, Master's in public health- Remove apostrophe. Is your degree pluralized?

Abstract: The phrase ‘’retailers nearby school’’ is ambiguous and seems to run through your prose. Please reconsider

Consider ‘’barriers to the implementation of legislation at the district level’’ in place of barriers to implement the legislation…

25: close to a school OR close to schools

28: Could your estimated aOR be considered insignificant considering the CI; Clarify why you consider this worthy of noting please

Also were there any other significant inferences you could state to buttress your other findings?

39: found to be…

42 and school level students are growing of 42 cigarette smoking; Reconsider this phrase

Also the whole sentence is verbose and ambiguous. Kindly restructure

49: Reconsider scientifically

51, 52: Please justify with examples what exactly the tobacco companies have done with regards to interference with legislation?

55: ‘this study’ to replace ‘the current study’

56: Consider omitting and they

57: the district level

62, 63: Any reason your sites are in brackets?

64: You are missing a period

66: …and school administrators…

69, 70: Restructure sentence

79: remove according

80: Either examined by experts or experts were consulted; Rephrase sentence

97 Review grammar. Only 3 provisions those are directly?

112. Review …and most of them selling…

113 …were conviced…

116: Despite of that? Review please

120: See above

124: Apply above in a whole prose

137-140: Refer to comment 28; Also why was the odds and CI not stated for the other finding?

146: …properly implement…; Also review the whole sentence for grammatical errors

155,157: The examples you have cited as per countries do not substantiate that the problem is more severe in developing countries. India is still a developing country albeit a rapidly growing economy?

160: small amount? Reconsider please

161: one of the cities…

Generally a promising addition to the knowledge database, however requires meticulous fine-tuning.
